# Supplementary material for: Sleep-Disturbance-Induced Microglial Activation Involves CRH-Mediated Galectin 3 and Autophagy Dysregulation
Source: Cells. 2022 Dec 30;12(1):160. doi: 10.3390/cells12010160 (PMC9818437; doi:10.3390/cells12010160)
Supplement: Supplementary file 1 [file cells-12-00160-s001.zip › cells-2097672-supplementary.pdf]

Supplementary materials

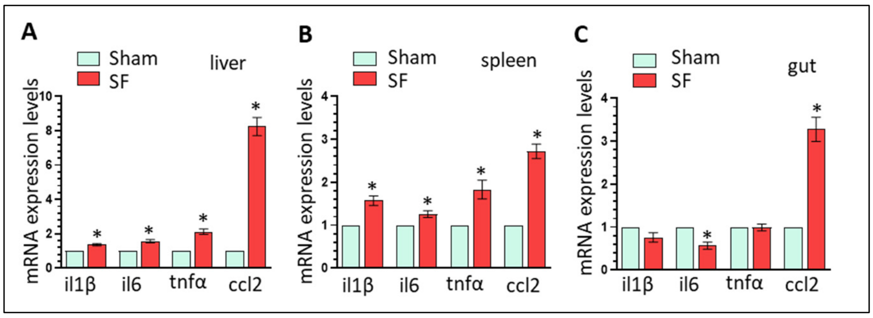

Supplementary Figure S1: Three-weeks of SF increases liver, spleen and gut inflammation levels (* P < 0.05, sham vs. SF, n = 6). (S1A): Three-weeks of SF increases il1β, il6, tnfα, and ccl2 in the liver; (S1B): Three-weeks of SF increases il1β, il6, tnfα, and ccl2 in the spleen; (S1C): Three-weeks of SF increases ccl2 levels in the gut.

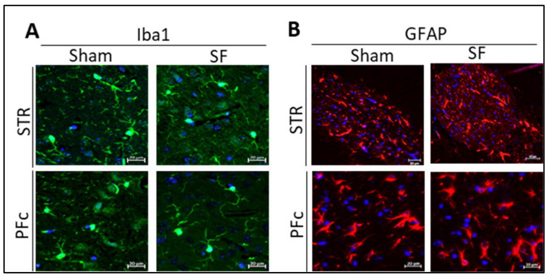

Supplementary Figure S2: Three-weeks of SF does not increase signaling intensity of Iba and GFAP in the striatum and PFc (scale bar = 20 μ). (S2A): No significant difference on Iba1 intensity in the striatum and PFc between sham and SF groups; (S2B): No significant difference on GFAP intensity in the striatum and PFc between sham and SF groups;

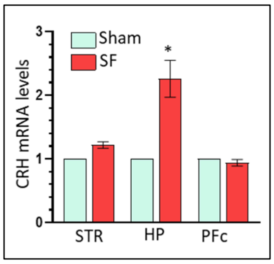

Supplementary Figure S3: Three-weeks of SF increases CRH mRNA levels in the HP but not in the striatum and PFc (* P < 0.05).

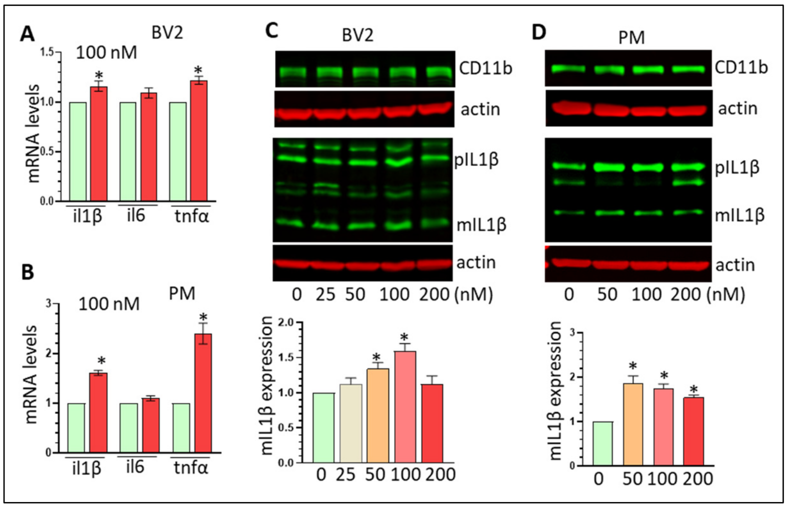

Supplementary Figure S4: CRH activates Mg *in vitro*. **(A, B):** CRH increases il1β and tnfα in both BV2 cells and PM (* P < 0.05); (**C**): CRH increases CD11b and mIL1β in BV2 cells (* P < 0.05); (**D**): CRH increases CD11b and mIL1β in PM (* P < 0.05).

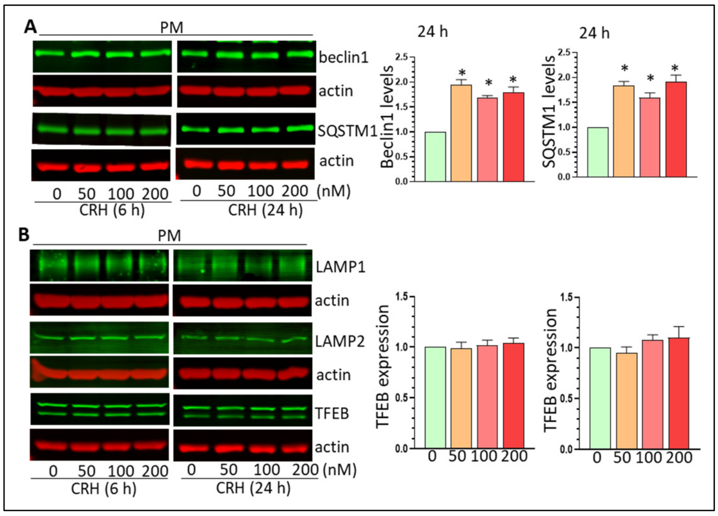

Supplementary Figure S5: The effects of CRH on autophagy processes. (**A**): CRH increases beclin1 and SQTM1 levels in PM after 24 hours treatment (* P < 0.05); (**B**): CRH has no effects on the levels of LAMP1/2 and TFEB in PM in PM at 6 and 24 hours after treatment.

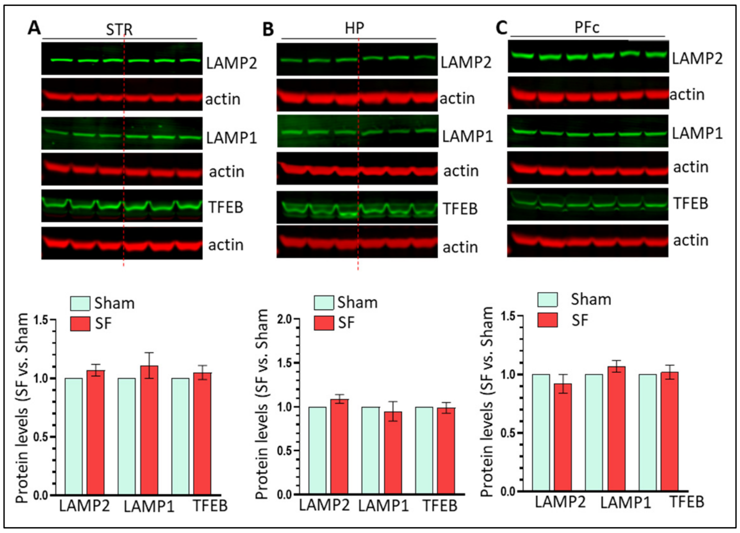

Supplementary Figure S6: The effects of three weeks of SF on lysosomal biogenesis in the brain. **(A):** Three weeks of SF had no effects on the levels of LAMP1/2, TFEB in the striatum; (**B**) Three weeks of SF had no effects on the levels of LAMP1/2, TFEB in the HP; (**C**): Three weeks of SF had no effects on the levels of LAMP1/2, TFEB in the PFc.
